# Role of peroxiredoxin of the AhpC/TSA family in antioxidant defense mechanisms of *Francisella tularensis*

**DOI:** 10.1371/journal.pone.0213699

**Published:** 2019-03-14

**Authors:** Arwa Alharbi, Seham M. Rabadi, Maha Alqahtani, Dina Marghani, Madeline Worden, Zhuo Ma, Meenakshi Malik, Chandra Shekhar Bakshi

**Affiliations:** 1 Department of Microbiology and Immunology, New York Medical College, Valhalla, New York, United States of America; 2 Department of Basic and Clinical Sciences, Albany College of Pharmacy and Health Sciences, Albany, New York, United States of America; East Carolina University Brody School of Medicine, UNITED STATES

## Abstract

*Francisella tularensis* is a Gram-negative, facultative intracellular pathogen and the causative agent of a lethal human disease known as tularemia. Due to its extremely high virulence and potential to be used as a bioterror agent, *F*. *tularensis* is classified by the CDC as a Category A Select Agent. As an intracellular pathogen, *F*. *tularensis* during its intracellular residence encounters a number of oxidative and nitrosative stresses. The roles of the primary antioxidant enzymes SodB, SodC and KatG in oxidative stress resistance and virulence of *F*. *tularensis* live vaccine strain (LVS) have been characterized in previous studies. However, very fragmentary information is available regarding the role of peroxiredoxin of the AhpC/TSA family (annotated as AhpC) of *F*. *tularensis* SchuS4; whereas the role of AhpC of *F*. *tularensis* LVS in tularemia pathogenesis is not known. This study was undertaken to exhaustively investigate the role of AhpC in oxidative stress resistance of *F*. *tularensis* LVS and SchuS4. We report that AhpC of *F*. *tularensis* LVS confers resistance against a wide range of reactive oxygen and nitrogen species, and serves as a virulence factor. In highly virulent *F*. *tularensis* SchuS4 strain, AhpC serves as a key antioxidant enzyme and contributes to its robust oxidative and nitrosative stress resistance, and intramacrophage survival. We also demonstrate that there is functional redundancy among primary antioxidant enzymes AhpC, SodC, and KatG of *F*. *tularensis* SchuS4. Collectively, this study highlights the differences in antioxidant defense mechanisms of *F*. *tularensis* LVS and SchuS4.

## Introduction

*Francisella tularensis* is a Gram-negative, facultative intracellular pathogen and the causative agent of a lethal human disease known as tularemia. *F*. *tularensis* has a very broad host range and can infect a wide range of ticks, arthropods, and mammals [1]. *F*. *tularensis* subsp. *tularensis* (Type A) cause lethal tularemia in North America. The strains belonging to *F*. *tularensis* subsp. *holarctica* (Type B) are less infectious than the Type A strains and are prevalent throughout the Northern hemisphere. *F*. *novicida* has been classified as a separate subspecies and is known to cause infection in immunocompromised individuals [2]. Owing to its extremely high virulence and potential to be used as a bioterror agent, *F*. *tularensis* is classified by the CDC as a Category A Select Agent.

As an intracellular pathogen, *F*. *tularensis* infects a wide variety of phagocytic cells such as macrophages, neutrophils, dendritic cells and non-phagocytic cells, such as hepatocytes, erythrocytes, and epithelial cells. Macrophages serve as the major reservoir for *F*. *tularensis* [3,4]. Phagocytic cells including macrophages produce reactive oxygen and nitrogen species (ROS/RNS) in response to *Francisella* infection. To counter these, *F*. *tularensis* genome encodes a full repertoire of primary antioxidant enzymes. *Francisella* encodes two superoxide dismutases (Sods); an iron-containing SodB (FeSod) and a copper-zinc containing SodC (CuZnSod) for the dismutation of superoxide radicals into hydrogen peroxide (H_2_O_2_). SodB is secreted via a major facilitator superfamily (MFS) type Emr multidrug efflux pump extracellularly [5] or in the cytosol of the infected macrophages [6]. A point mutant of the *sodB* gene is hypersensitive to oxidative stress and attenuated for virulence in mice [7]. SodC of *Francisella* is located in the periplasm of the bacterial cell and is required for resistance from extracellularly generated oxidative stress, and virulence in mice [8]. A catalase encoded by the *katG* gene converts H_2_O_2_ in water and oxygen and therefore prevents generation of other microbicidal ROS such as hydroxyl (HO^.^) radicals or hypochlorous acids (HOCl), and thus play an important role in resistance of *Francisella* against oxidative stress. KatG similar to SodB is secreted in the extracellular environment or the macrophage cytosol [5,9]. The *katG* gene deletion mutants (Δ*katG*) of both *F*. *tularensis* subspecies *holarctica* Live Vaccine Strain (LVS) and the highly virulent *F*. *tularensis* subspecies *tularensis* SchuS4 strain are sensitive to H_2_O_2_, but not to RNS, peroxynitrite (ONOO^-^). The Δ*katG* mutant of both the LVS and SchuS4 replicate similar to their respective wild type strains in unstimulated macrophages. However, only the Δ*katG* mutant of *F*. *tularensis* LVS is attenuated for virulence; while the SchuS4 Δ*katG* mutant remains virulent in mice [10,11]. In addition to these primary antioxidant enzymes, *Francisella* also encodes glutathione peroxidase (Gpx), MoxR ATPases, Dyp-type Peroxidase, glutaredoxin A (GrxA), and methionine sulfoxide reductase A, A1 and B [12]. A MoxR subfamily protein encoded by *FTL_0200* gene of *F*. *tularensis* LVS provides resistance against oxidative and pH stresses [13]. A gene encoding a protein with sequence similarity to organic hydroperoxide resistance protein Ohr found in several bacterial pathogens is also reported in *F*. *novicida* and *F*. *tularensis* LVS. This *ohr* homolog is required for resistance against organic peroxides as well as NADPH-generated ROS both *in vitro* and *in vivo* [4]. It has been reported that *FTT_0086* of *F*. *tularensis* SchuS4 is required for resistance against oxidative stress; while a homolog of this gene is not functional in *F*. *tularensis* LVS [10]. Collectively, these studies demonstrate that differences do exist between the antioxidant defenses of *F*. *tularensis* LVS and *F*. *tularensis* SchuS4.

A highly conserved LysR family of regulators known as OxyR is also present in *F*. *tularensis*. H_2_O_2_ activates OxyR via the modification of an oxidant-sensitive cysteine residue which then binds to the promoter region of the target genes and upregulates their expression. In our previous study, we have demonstrated that OxyR regulates the expression of antioxidant enzyme genes alkyl hydroperoxide reductase (*ahpC*) and *katG* [14]. The AhpC belongs to a family of thiol peroxidases (peroxiredoxins) that can scavenge micromolar concentrations of H_2_O_2_. The catalases are activated only after AhpC is saturated with millimolar concentrations of H_2_O_2_. AhpC uses cysteine thiols to reduce peroxides and acts in conjunction with AhpC reductants; AhpF or AhpD, that recycle AhpC during catalysis [15]. Both these reductants are absent in *F*. *tularensis*. The open reading frames *FTL_1015* in *F*. *tularensis* LVS and *FTT_0557* in *F*. *tularensis* SchuS4 do not code for AhpC proteins, instead they are more structurally similar to peroxidase/peroxireducatse proteins and has been annotated as peroxiredoxin of the AhpC/TSA family. It has been reported that AhpC in *F*. *tularensis* SchuS4 is required for resistance against endogenous H_2_O_2_ and ONOO^-^ [10]. However, very fragmentary information is available regarding the role of AhpC of *F*. *tularensis* SchuS4 in the pathogenesis of tularemia [10,16]; whereas the role of AhpC of *F*. *tularensis* LVS is not known. This study was undertaken to exhaustively investigate the role of AhpC in oxidative stress resistance of *F*. *tularensis* LVS and SchuS4. We report that AhpC of *F*. *tularensis* LVS confers resistance against a wide range of ROS and RNS, and serves as a virulence factor. This study also demonstrates that there is a functional redundancy among primary antioxidant enzymes AhpC, KatG and SodC of *F*. *tularensis* SchuS4. However, AhpC serves as a key antioxidant enzyme and contributes to robust oxidative and nitrosative stress resistance and intramacrophage survival of the highly virulent *F*. *tularensis* SchuS4 strain.

## Materials and methods

### Ethics statement

This study was carried out in strict accordance with the recommendations and guidelines of the National Council for Research (NCR) for care and use of animals. All the animal experiments were conducted in the centralized Animal Resources Facility of New York Medical College licensed by the USDA and the NYS Department of Health, Division of Laboratories and Research and accredited by the American Association for the Accreditation of Laboratory Care. The use of animals and protocols were approved by the Institutional Animal Care and Use Committee (IACUC) of New York Medical College (Protocol Number 69-2-0914H). Mice were administered an anesthetic cocktail consisting of ketamine (5 mg/kg) and xylazine (4 mg/kg) and underwent experimental manipulation only after they failed to exhibit a toe pinch reflex. Mice exhibiting more than 25% weight loss, anorexia, dehydration and impairment of mobility were removed from the study and euthanized by approved means. Humane endpoints were also necessary for mice which survived at the conclusion of the experiments. Mice were administered an anesthetic cocktail of ketamine and xylazine intraperitoneally and then euthanized via cervical dislocation followed by cardiac puncture, a method that is consistent with recommendations of the Panel on Euthanasia of the American Veterinary Medical Association. In all experimental procedures, efforts were made to minimize pain and suffering. All the work with Category A select agent *F*. *tularensis* SchuS4 was performed in CDC Certified Biosafety Level 3 (BSL3) laboratory of New York Medical College (Registration No. C20160722-1812) in accordance with protocols approved by Institutional Biosafety Committee (Protocol No. 01-2015-3).

### Bacterial strains and growth conditions

*F*. *tularensis* subspecies *holarctica* LVS and *F*. *tularensis* subspecies *tularensis* SchuS4 used in this study were obtained from BEI Resources (Manassas, VA). The *ahpC* (*FTL_1015*) gene deletion (Δ*ahpC*) mutant of *F*. *tularensis* LVS and a transcomplemented strain (Δ*ahpC*+p*ahpC*) were generated and used in this study. Previously published Δ*sodC* mutant of *F*. *tularensis* LVS available in our laboratory was also used in this study (8). The gene deletion mutants of *F*. *tularensis* SchuS4; Δ*ahpC* (*FTT_0557*), Δ*katG* (*FTT_0721c*) and the Δ*sodC* (*FTT_0879*), and *F*. *tularensis* LVS Δ*katG* (11) mutants were kindly provided by Dr. Andres Sjostedt (Umea University, Sweden). All the bacterial strains used in this study are shown in Table 1. All the experiments involving *F*. *tularensis* SchuS4 strain were conducted in the CDC certified BSL3 laboratory of New York Medical College.

**Table 1:** List of bacterial strains and plasmids used in this study.

Strains	Genotype	Source
*Francisella tularensis* LVS	Wild type strain	BEI Resources
Δ*ahpC* mutant	Deletion mutant of *F*. *tularensis* LVS *ahpC* gen	This study
*ahpC* transcomplement (Δ*ahpC* + p*ahpC*)	*F*. *tularensis* LVS, Δ*ahpC*, pMM09 (pMP822+*ahpC*), Hygro^r^	This study
Δ*sodC* mutant	Deletion mutant of *F*. *tularensis* LVS *sodC* gene	[8]
Δ*katG* mutant	Deletion mutant of *F*. *tularensis* LVS *katG* gene	[11]
*Francisella tularensis* SchuS4	Wild type strain	BEI Resources
*F*. *tularensis* SchuS4 Δ*ahpC* mutant	Deletion mutant of *F*. *tularensis* SchuS4 *ahpC* gene	[10]
*F*. *tularensis* SchuS4 Δ*sodC* mutant	Deletion mutant of *F*. *tularensis* SchuS4 *sodC* gene	[16]
*F*. *tularensis* SchuS4 Δ*katG* mutant	Deletion mutant of *F*. *tularensis* SchuS4 *katG* gene	[11]
*E*. *coli* DH5α	F– Φ80*lac*ZΔM15 Δ(*lac*ZYA-*arg*F) U169 *rec*A1 *end*A1 *hsd*R17 (rK–, mK+) *pho*A *sup*E44 λ– *thi*-1 *gyr*A96 *rel*A1	Invitrogen
**Plasmids**		
pMP822	*E*. *coli-Francisella* shuttle vector, Hygro^r^	[17]
pJC84	*E*. *coli-Francisella* suicide vector, Kan^r^	[12]
pMM06	pJC84 + fused flanking fragment of *ahpC* gene, Kan^r^	This study

All bacterial strains were grown on Mueller-Hinton (MH)-chocolate agar plates (BD Biosciences, San Jose, CA) at 37°C with 5% CO_2_ or Muller-Hinton broth (MHB) (BD Biosciences, San Jose, CA) supplemented with IsoVitaleX and ferric pyrophosphate at 37°C with constant shaking (175 rpm). Transcomplemented Δ*ahpC*+p*ahpC* strain of *F*. *tularensis* LVS was grown on MH-chocolate agar plates supplemented with hygromycin (200μg/mL). Bacterial strains were grown in MHB to mid-log phase, aliquoted and stored at -80°C until further use.

### Construction of Δ*ahpC* mutant and transcomplementation

Allelic replacement method was used to construct the Δ*ahpC* mutant of *F*. *tularensis* LVS [18]. The entire 557-bp coding region of the *ahpC* gene (*FTL_1015*) was deleted employing an approach described previously [19]. Briefly, a 5’ 1218 bp fragment upstream of the start codon and first 5 bp of the *ahpC* gene was amplified with primers MP241 and 243. A 3’ fragment containing last 10 bp and the stop codon of the *ahpC* gene and 1218 bp of the downstream region was amplified with primers MP245 and 246. Both the upstream and downstream fragments were joined by overlapping extension PCR with primers MP241 and MP246 engineered with *Bam*HI and *Sal*I restriction sites at 5’ and 3’ ends, respectively. The generated single fragment with *ahpC* gene deletion was digested and cloned into the pJC84 vector using the *Bam*HI and *Sal*I sites. The resultant plasmid, pMM06, was electroporated into the wild type *F*. *tularensis* LVS as described previously [14,20]. After the primary selection of positive colonies using kanamycin and a counter selection with sucrose, the positive colonies were screened by colony PCR with primers MP260 and MP261 to identify the Δ*ahpC* mutant.

For transcomplementation of the Δ*ahpC* mutant of *F*. *tularensis* LVS, full-length *ahpC* gene sequence was amplified with primers MP274 and MP275 and cloned into a pMP822 vector at *Bam*HI site generating a plasmid, pMM09. The pMM09 plasmid was transformed into chemically competent *E*. *coli* DH5α cells and selected on LB-hygromycin plates. The pMM09 was purified, and the orientation of the *ahpC* gene in the pMM09 vector was confirmed by PCR. The pMM09 vector cloned in the correct orientation was electroporated in the Δ*ahpC* mutant. The transformants were selected on MH-chocolate agar plates containing hygromycin (200μg/mL). The resultant transcomplemented strain was termed as Δ*ahpC*+p*ahpC* and confirmed by PCR. The primer sequences and the vectors used for the generation of Δ*ahpC* and Δ*ahpC*+p*ahpC* strains are shown in Table 2.

**Table 2:** List of primer sequences used in this study.

Primer	Sequence	Purpose
***ahpC* gene deletion construct:**		
***F*. *tularensis* LVS *ahpC* upstream fragment**		
MP241*	5’-CAAggatccTCCATTTGCAGAGGCTTTTG -3’	Forward primer with a *BamH*I site
MP243	5’-CCTTTTCATAATTACTTAGACTCTGTCATGTCTAACT CCTTTGTTTTG-3’	Reverse-primer
***F*. *tularensis* LVS *ahpC* gene downstream fragment**		
MP245	5’-CAAAACAAAGGAGTTAGACATGACAGAGTCTAAGT AATTATGAAAAGG-3’	Forward primer
MP246*	5’-tgatgtcgacGACTAGCTGCCCTACACTGTTTTA-3’	Reverse primer with a *Sal*I site
***F*. *tularensis*** Δ***ahpC* mutant screening**		
MP260	5’-AATGCAGGTTGGCTGACAAA-3’	Forward primer for *ahpC*
MP261	5’-CGCCAGAAAAACTTACAGTTACTA-3’	Reverse primer for *ahpC*
**Transcomplementation construct**		
**For transcomplementation of *F*. *tularensis* LVS** Δ***ahpC* mutant**		
MP274*	5’-CAAggatccATGACTAAAAAAGTACCTAATGT-3’	Forward primer for *ahpC* with a *BamH*I site
MP275*	5’-TGATctcgagTTACTTAGACTCTAAATACTTCAA-3’	Reverse primer for *ahpC* with an *Xho*I site

*Underlined lower case letters denote the restriction enzyme site.

### Growth curves

Growth curves were generated by resuspending bacterial cultures grown on MH-chocolate agar plates to an Optical Density at 600 nm (OD_600_) of 0.2 (corresponds to 1×10^9^ CFU/mL) in MHB. The bacterial suspensions were grown for 28 hours in the absence or presence of 750μM H_2_O_2,_ and the OD_600_ was recorded at 4-hour intervals.

### Disc diffusion assays

Disc diffusion assays were used to determine the sensitivity of *F*. *tularensis* LVS, and *F*. *tularensis* SchuS4 strains towards superoxide-generating compounds, organic peroxides, and H_2_O_2_. Cultures of wild type *F*. *tularensis* LVS, the Δ*ahpC* mutant and the Δ*ahpC*+p*ahpC* transcomplemented strain grown on MH-chocolate agar plates were resuspended in 1mL of sterile PBS and adjusted to an OD_600_ of 2.0. The suspensions were then spread on MH-chocolate agar plates using sterile cotton swabs to obtain a heavy bacterial growth. Sterile filter paper discs were impregnated with 10 μL of varying concentrations of superoxide-generating compounds; menadione (1.56 μg/disc), pyrogallol (62.5 μg/ disc), and paraquat (3.75 μg/disc) (Sigma Aldrich, St. Louis, MO) as well as organic peroxides *tert*-butyl hydroperoxide (TBH) (437μg/disc), cumene hydroperoxide (CHP) (125 μg/ disc), and H_2_O_2_ (6.25 mM/disc) (Sigma Aldrich, St. Louis, MO). An identical protocol was used for disc diffusion assays performed with wild type *F*. *tularensis* SchuS4 and the Δ*ahpC*, Δ*sodC*, and Δ*katG* mutants. However, higher concentrations of oxidants than those used for *F*. *tularensis* LVS were used. Specifically, the concentrations of menadione (6.25μg/disc), paraquat (15μg/disc) and pyrogallol (250 and 500μg/disc); organic peroxides TBH (3.5mg/disc); CHP (500μg/disc); and H_2_O_2_ (50 mM/disc) (Sigma Aldrich, St. Louis, MO) were used. The plates were incubated at 37°C in the presence of 5% CO_2_ for 48 hours. The zone of inhibition around the discs was measured in millimeters (mm).

### Spot assays

Spot assays were performed to determine the sensitivities of *F*. *tularensis* LVS, the Δ*ahpC* mutant and the Δ*ahpC*+p*ahpC* toward superoxide-generating compounds, peroxides, and RNS generating compounds. Serial two-fold dilutions of superoxide-generating compound menadione (starting concentration 62.5μg), pyrogallol (155μg), paraquat (155μg), TBH (34.7μg), CHP (27.4 μg), H_2_O_2_ (4.4mM), sodium nitroprusside (SNP) (375μg) (Ricca Chemical Company, Arlington, TX) and Sin-1 (0.5μg) (EMD Millipore corporation, Temecula, CA) were made in a sterile flat bottom 96-well plate in 100μL volume of MHB. The Δ*sodC* and Δ*katG* mutants of *F*. *tularensis* LVS were also tested using similar concentrations of menadione, TBH and CHP. The bacterial suspensions of *F*. *tularensis* LVS, Δ*ahpC* mutant, and the Δ*ahpC*+p*ahpC* cultures grown on MH chocolate agar plate were resuspended in MHB and adjusted to an OD_600_ of 0.2. 100μL of bacterial suspensions were added to each well and mixed. The *F*. *tularensis* SchuS4 and SchuS4 Δ*ahpC*, Δ*sodC* and Δ*katG* mutants were exposed to 2-fold diluted menadione (Starting concentration 12.5 μg), TBH (875 μg), CHP (62.5 μg), SNP (15.7μg) and Sin-1 (12.5μg) to test their sensitivities towards these compounds. The plates were incubated at 37°C in the presence of 5% CO_2_ for 1 and 3 hours post-exposure, and 3μL bacterial cultures from each dilution were spotted on MH-chocolate agar plates using a multichannel pipette. The sensitivity to the compounds tested was determined on the basis of observable growth pattern on the plates after 48 hours of incubation.

### Cell culture assays

A murine macrophage cell line Raw264.7 was used in cell culture-based assays. The macrophages were infected with the wild-type *F*. *tularensis* LVS, the Δ*ahpC* mutant, and the Δ*ahpC*+p*ahpC* transcomplemented strain at a multiplicity of infection (MOI) of 10 and 100 in a volume of 1 mL bacterial suspension. In separate experiments, Raw264.7 macrophages were infected with the wild-type *F*. *tularensis* SchuS4, the Δ*ahpC*, Δ*sodC*, and Δ*katG* mutants at an MOI of 100 as described previously [5,14]. The infected cells were lysed after 4 and 24 hours of infection with 0.1% sodium deoxycholate, diluted 10-fold in sterile PBS and plated on MH-chocolate agar plates. The plates were incubated at 37°C in the presence of 5% of CO_2_ for 48 hours, and the colonies were counted. Results were expressed as Mean ± SD of three biological replicates and presented as Log_10_ colony forming units (CFU)/mL.

### Mouse challenge studies

All mice studies followed the protocols approved by the Institutional Animal Care and Use Committee (IACUC) of New York Medical College. Six to eight-week-old wild type C57BL/6 and gp91*phox*^*-/-*^ mice were obtained from Jackson Laboratories. Mice were maintained in a pathogen-free environment in the Animal Facility of New York Medical College (Valhalla, NY) Mice (n = 4 mice/group) were deeply anesthetized by intraperitoneal injection of Ketamine/Xylazine cocktail. The loss of reflexes in anesthetized mice was determined by the loss of toe-pinch reflex. The deeply anesthetized mice were inoculated intranasally with 1x10^4^ CFU of the wild-type *F*. *tularensis* LVS strain or the Δ*ahpC* mutant resuspended in 20μL PBS (10μL/nare). The infected mice were observed for morbidity and mortality for 21 days. The survival results were plotted as Kaplan-Meier survival curves, and the data were analyzed statistically by the Log-rank test.

### Statistical analysis

Statistical analysis was performed by using GraphPad Prism and InStat software. The results were expressed as Mean ± S.E.M. or S.D., and statistical significance between groups was determined by one-way ANOVA followed by Bonferroni’s corrections or student t-test. As detailed earlier, the survival results were expressed as Kaplan-Meier survival curves, and *P* values were determined by the Log-rank test.

## Results

**Fig 1:**
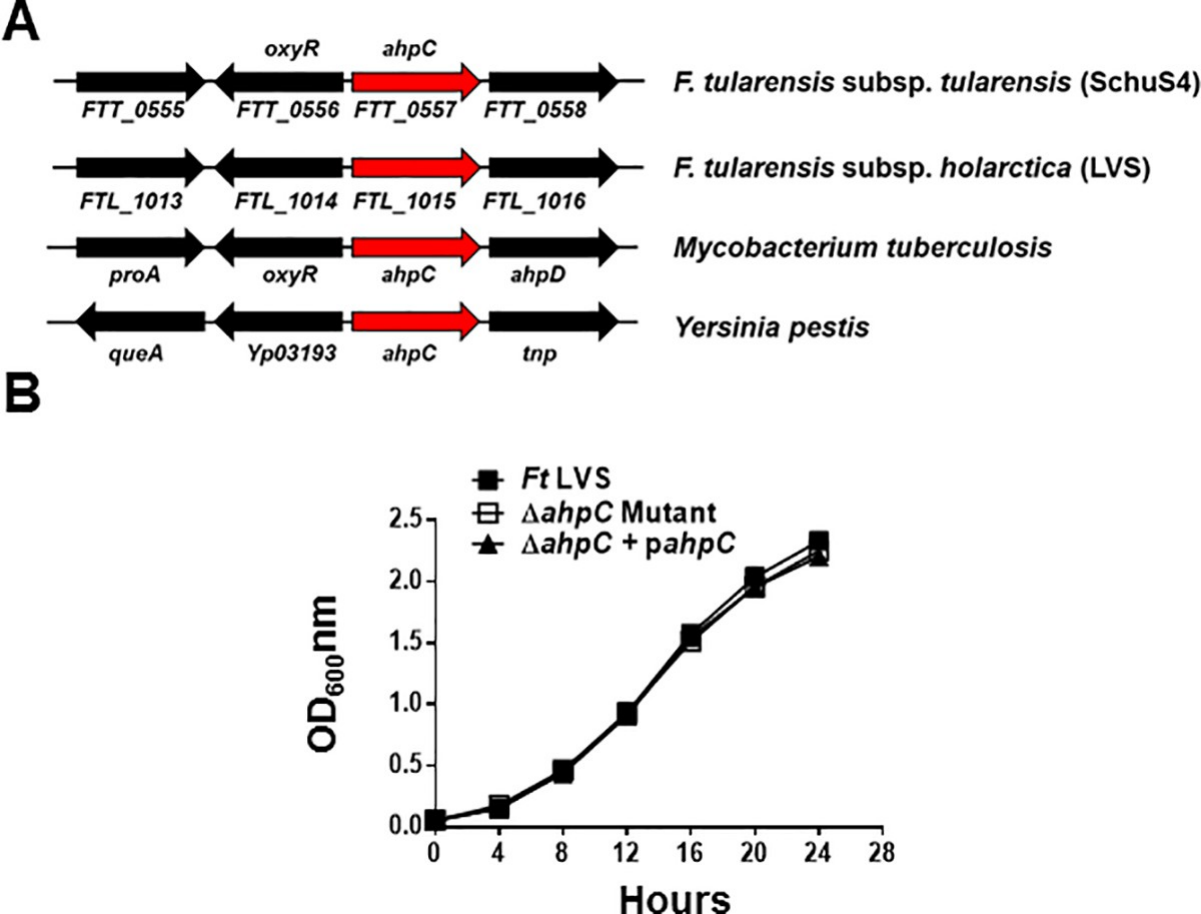
Genomic organization and growth characteristics of the Δ*ahpC* mutant of *F*. *tularensis* LVS. (A) Genomic organization of the *ahpC* gene. (B) Growth curves of *F*. *tularensis* LVS, the Δ*ahpC* mutant and the transcomplemented strains (Δ*ahpC* + p*ahpC*). Equal numbers of bacteria were suspended in Mueller-Hinton broth, and the optical densities (OD_600_) were recorded every 4 hours.

The peroxiredoxin of the AhpC/TSA family (*ahpC*) gene in *F*. *tularensis* LVS and SchuS4 is transcribed divergently from the LysR family oxidative stress transcriptional regulator gene, *oxyR*. A similar genomic organization of *ahpC* gene is also present in *Mycobacterium tuberculosis*. However, in other bacterial pathogens including *Yersinia pestis*, the *ahpC* gene is not transcribed divergently from the *oxyR* gene (Fig 1A). To characterize the functional role of the peroxiredoxin AhpC of *F*. *tularensis* LVS, we generated a gene deletion mutant of *ahpC* (Δ*ahpC*). The deletion of the *ahpC* gene was confirmed by PCR followed by DNA sequencing to determine that *ahpC* gene deletion did not alter reading frames of the downstream genes. A transcomplement of the Δ*ahpC* mutant was generated by providing a copy of *ahpC* gene in-trans. Transcomplementation was confirmed by PCR using *ahpC* gene-specific primers. The Δ*ahpC* mutant was tested for any growth defect under aerobic growth conditions. It was observed that growth pattern of the Δ*ahpC* mutant was identical to that of the wild type *F*. *tularensis* LVS or the transcomplemented strain when grown aerobically indicating that the loss of *ahpC* is not associated with any growth defect in the Δ*ahpC* mutant (Fig 1B).

### The Δ*ahpC* mutant of *F*. *tularensis* LVS exhibits enhanced sensitivities towards superoxide-generating compounds

**Fig 2:**
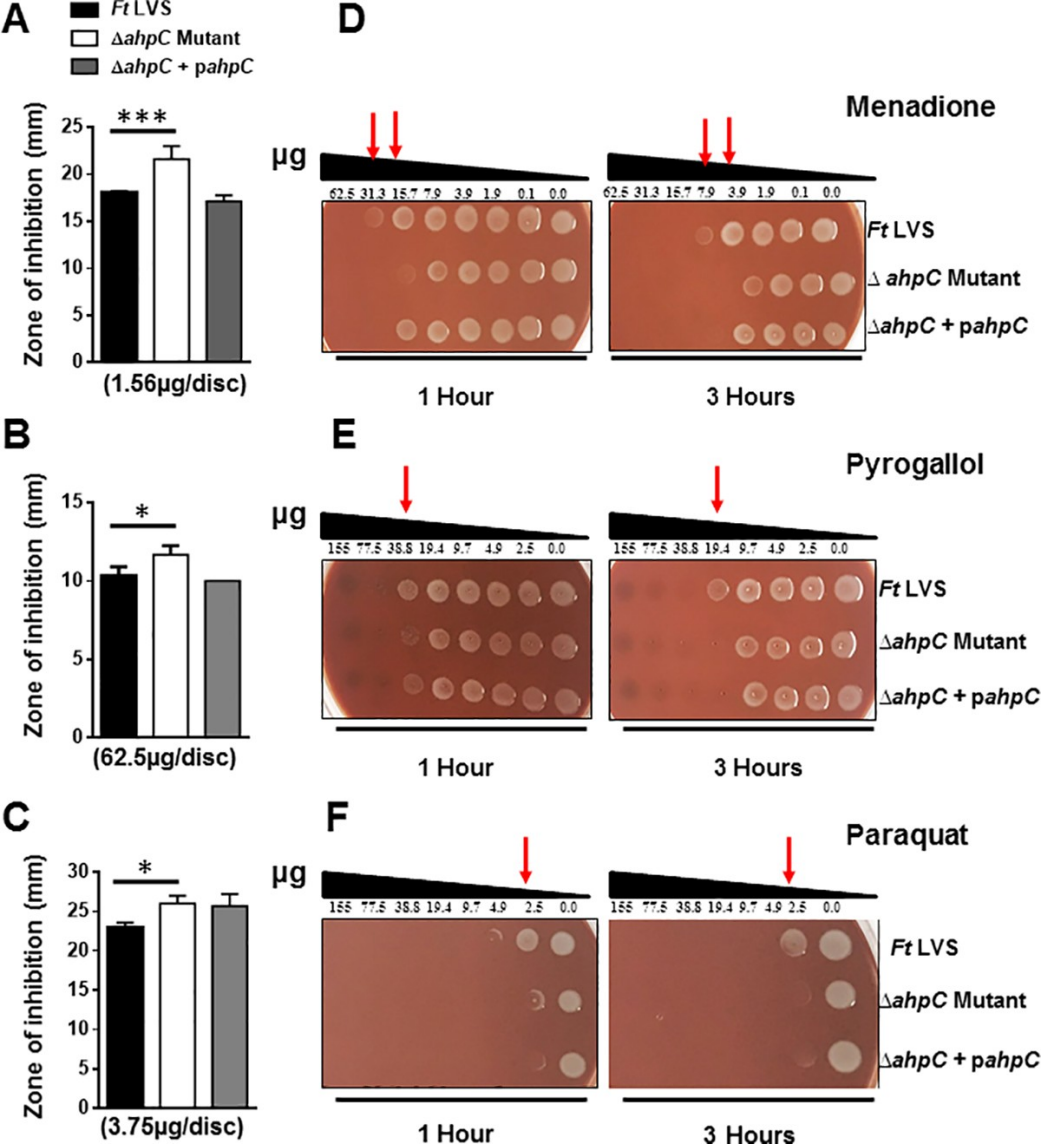
The Δ*ahpC* mutant of *F*. *tularensis* LVS exhibits enhanced sensitivities towards superoxide generating compounds. The sensitivities of the wild type *F*. *tularensis* (*Ft*) LVS, the Δ*ahpC* mutant, and the transcomplemented strain (Δ*ahpC*+p*ahpC*) as determined by disc diffusion and spot assays against superoxide-generating compounds menadione (A and D), pyrogallol (B and E) and paraquat (C and F**)**. For disc diffusion assays, the results are expressed as a zone of inhibition in millimeters obtained using the indicated concentrations of the compounds and are expressed as Mean ± S.D. of triplicate samples. In spot assays, the *Francisella* strains were exposed to serially diluted menadione, pyrogallol and paraquat for 1 and 3 hours and spotted on MH-chocolate agar plates to determine the bacterial killing. The red arrows indicate enhanced killing of the Δ*ahpC* mutant at the indicated concentrations of the compounds. All the results shown are representative of 3 independent experiments conducted. The *p* values were determined by one-way ANOVA and a *p*-value of <0.05 is considered statistically significant. *p<0.05; ***p<0.001.

The contribution of AhpC of *F*. *tularensis* LVS in conferring resistance to superoxide-generating compounds menadione, pyrogallol and paraquat were determined by disc diffusion and spot assays. The Δ*ahpC* mutant of *F*. *tularensis* LVS revealed enhanced sensitivities towards superoxide-generating compounds as indicated by significantly larger zones of inhibition around the discs impregnated with menadione (21.6±1.4 mm), pyrogallol (11.6±0.6 mm) and paraquat (26.0±1.0 mm) as compared to those observed for wild type *F*. *tularensis* LVS (18.0±0.2, 10.3±0.6, 23.0±1.0 mm, respectively) and the transcomplemented strain (17.1±0.6, 10.0±0.0, 25.6±1.5 mm, respectively) (Fig 2A, 2B and 2C). Similar to the Δ*ahpC* mutant, the Δ*sodC* and Δ*katG* mutants of *F*. *tularensis* LVS also exhibited increased susceptibility towards menadione (S1A and S1B Fig).

We next confirmed the results obtained with the disc diffusion assays by performing spot assays that determine the bacterial viability. Wild type *F*. *tularensis* LVS, the Δ*ahp*C mutant or the transcomplemented strains were exposed to varying concentrations of two-fold serial dilutions of menadione, pyrogallol and paraquat for 1 and 3 hours, and plated to determine the bacterial viability. Reduced viability of the Δ*ahpC* mutant was observed after 1 and 3 hours of exposure to menadione (31.25 and 15.62μg, respectively), pyrogallol (38.8 and 19.4μg, respectively) and paraquat (0.30 and 0.15μg, respectively) as compared to the wild type *F*. *tularensis* LVS corroborating the results observed with the disc diffusion assays. The transcomplementation either restored the wild type phenotype or exhibited an intermediate phenotype (Fig 2D, 2E and 2F). Collectively, these results demonstrate that loss of AhpC in *F*. *tularensis* LVS is associated with enhanced sensitivities towards the superoxide-generating compounds.

### The Δ*ahpC* mutant of *F*. *tularensis* LVS exhibits enhanced sensitivity towards organic peroxides and H_2_O_2_

**Fig 3:**
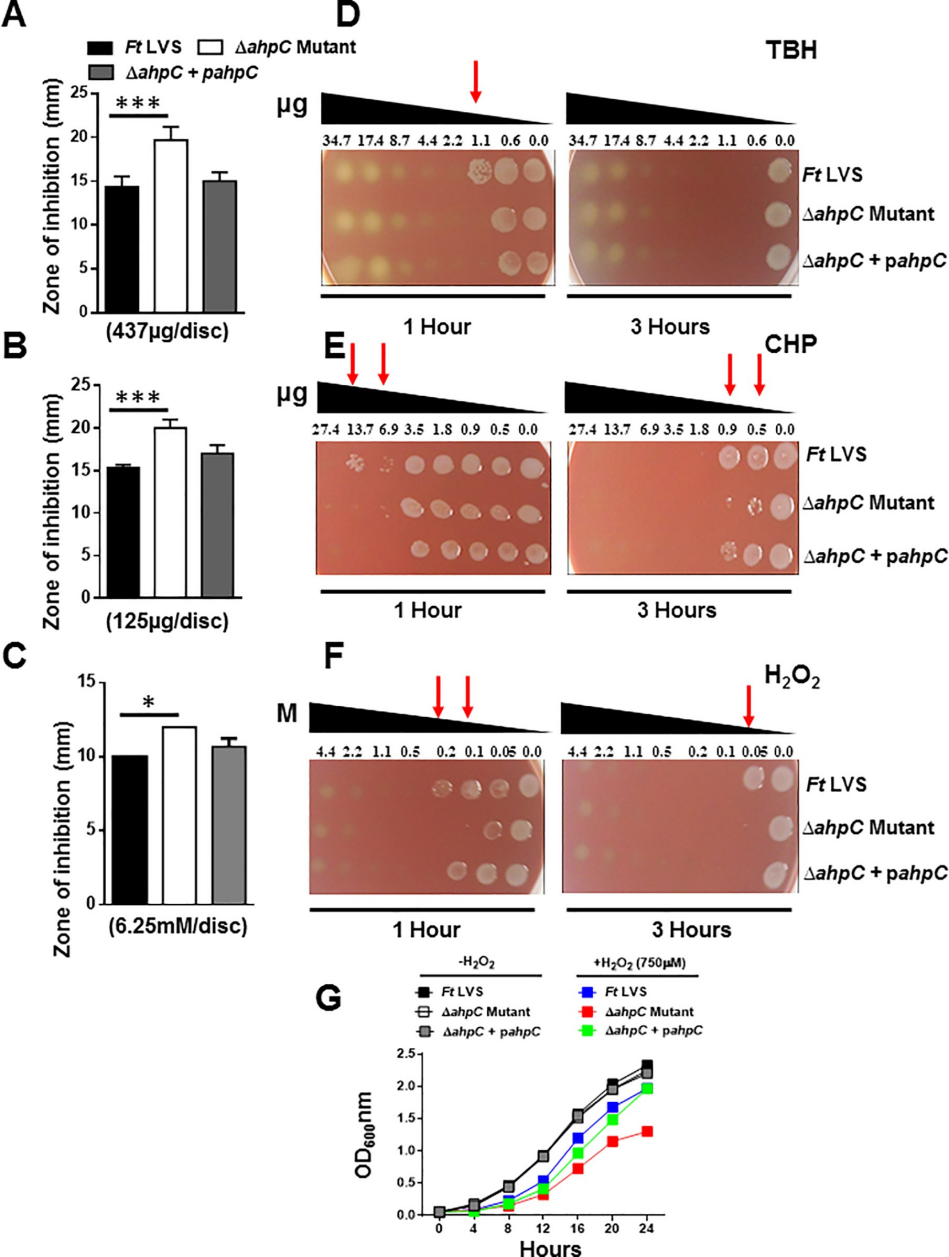
The Δ*ahpC* mutant of *F*. *tularensis* LVS exhibits enhanced sensitivity towards organic peroxides and H_2_O_2_. The sensitivities of the wild type *F*. *tularensis* (*Ft*) LVS, the Δ*ahpC* mutant, and the transcomplemented strain Δ*ahpC*+p*ahpC* as determined by disc diffusion and spot assays against organic peroxides tert-butyl hydroperoxide (TBH) (A and D), cumene hydroperoxide (CHP) (B and E) and H_2_O_2_ (C and F). For disc diffusion assays, the results are expressed as zone of inhibition in millimeters obtained using the indicated concentrations of the compounds and are expressed as Mean ± S.D. of triplicate samples. In spot assays, *Francisella* strains were exposed to serially diluted TBH, CHP, and H_2_O_2_ for 1 and 3 hours and spotted on MH-chocolate agar plates to determine the bacterial killing. The red arrows indicate enhanced killing of the Δ*ahpC* mutant at the indicated concentrations of the compounds. (G) Growth curves of *F*. *tularensis* LVS, the Δ*ahpC* mutant and the transcomplemented strain (Δ*ahpC* + p*ahpC*) in the absence or presence of 750μM H_2_O_2_. Equal numbers of bacteria were suspended in Mueller-Hinton broth and the optical density (OD_600_) was recorded every 4 hours. All the results shown are representative of 3 independent experiments conducted with identical results. The *p* values were determined by one-way ANOVA and a *p* value of <0.05 is considered statistically significant. *p<0.05; ***p<0.001.

The contribution of AhpC of *F*. *tularensis* LVS in conferring resistance to organic peroxides TBH and CHP, and H_2_O_2_ was determined by disc diffusion assay. The Δ*ahp*C mutant of *F*. *tularensis* LVS revealed enhanced sensitivities towards organic peroxides TBH (19.6±1.5 mm), CHP (20.0±1.0 mm) and H_2_O_2_ (12.0±0.0 mm) as observed by significantly larger zones of inhibition around the discs impregnated with these compounds as compared to those observed for the wild type *F*. *tularensis* LVS (14.3±2.0, 15.3±0.6, 10.0±0.0 mm, respectively) or the transcomplemented strain (15.0±1.0, 17.0±1.0, 10.6±0.5 mm, respectively) (Fig 3A, 3B, and 3C). Similar to the Δ*ahpC* mutant, the Δ*sodC* and Δ*katG* mutants of *F*. *tularensis* LVS also exhibited increased susceptibilities towards TBH and CHP (S1C, S1D, S1E and S1F Fig).

We confirmed the results obtained with the disc diffusion assays by performing spot assays and by generating growth curves in the presence of H_2_O_2_. The wild type *F*. *tularensis* LVS, the Δ*ahp*C mutant or the transcomplemented strains were exposed to varying concentrations of serially diluted TBH, CHP, and H_2_O_2_ for 1 and 3 hrs and plated to determine the bacterial viability. The viability of the Δ*ahp*C mutant of *F*. *tularensis* LVS was markedly reduced after 1 and 3 hours of exposure to TBH (1.1μg), CHP (13.7 and 0.9μg, respectively) and H_2_O_2_ (0.2 and 0.05mM, respectively) as compared to the wild-type *F*. *tularensis* LVS. Transcomplementation of the Δ*ahp*C mutant restored the wild type phenotype (Fig 3D, 3E and 3F). The Δ*ahp*C mutant grew very slowly as compared to the wild type or the transcomplemented counterparts when grown in the presence of 750μM of H_2_O_2_ (Fig 3G). Collectively, these results demonstrate that AhpC of *F*. *tularensis* LVS plays an important role in providing resistance against organic peroxides and H_2_O_2_.

### The Δ*ahpC* mutant of *F*. *tularensis* LVS exhibits enhanced sensitivity towards RNS

**Fig 4:**
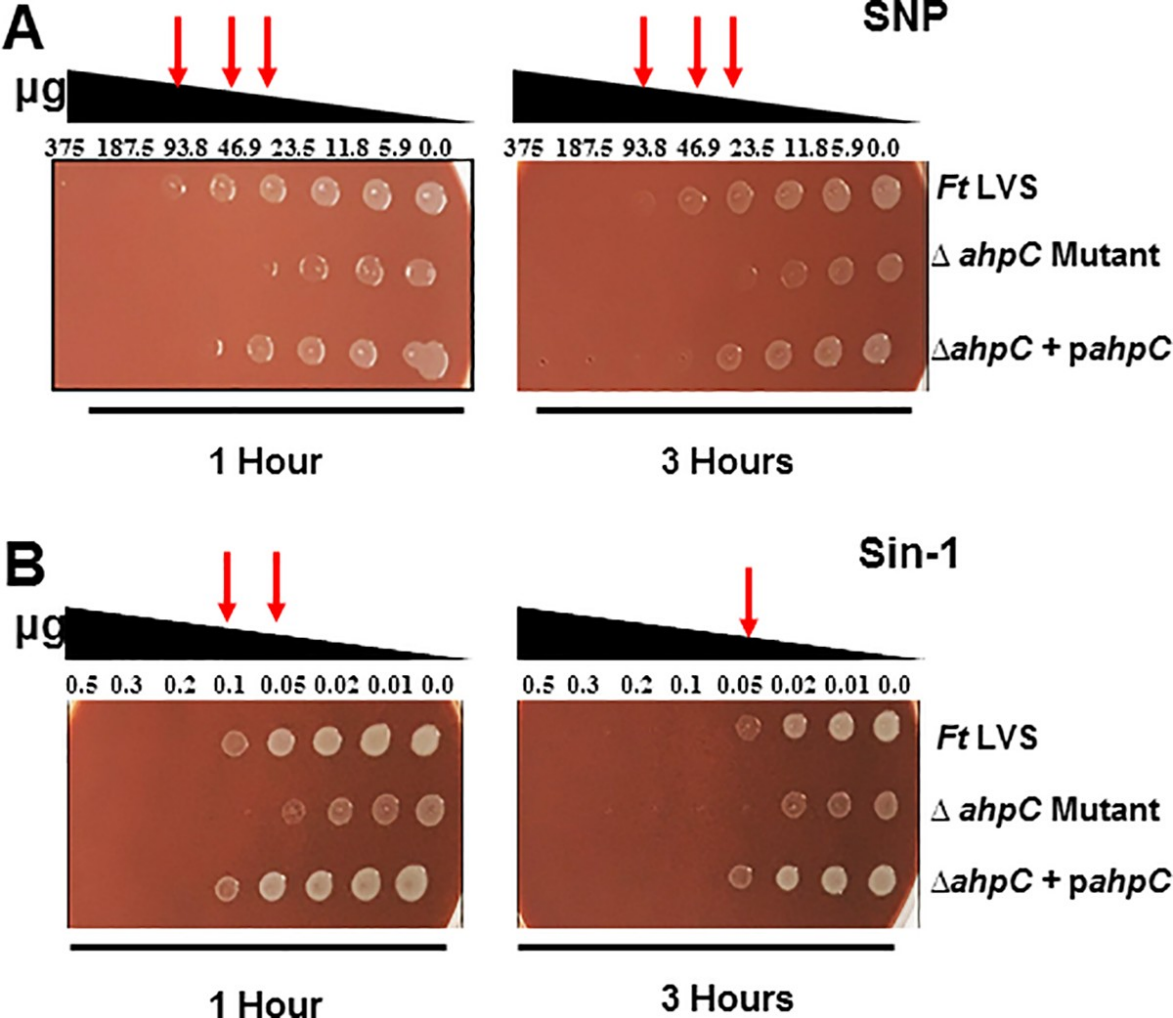
The Δ*ahpC* mutant of *F*. *tularensis* LVS exhibits enhanced sensitivity towards RNS. Spot assays were performed using nitric oxide (NO) donors (A) SNP and (B) Sin-1. Wild type *F*. *tularensis* LVS, the Δ*ahpC* mutant and the transcomplemented strains (Δ*ahpC* + p*ahpC*) were exposed to serially diluted compounds for 1 and 3 hours and spotted on MH-chocolate agar plates. The red arrows indicate enhanced killing of the Δ*ahpC* mutant at the indicated concentrations of the compounds. The results shown are representative of 3 independent experiments conducted.

Our preceding results demonstrated that AhpC of *F*. *tularensis* LVS provides resistance against superoxide-generating compounds and peroxides. We further tested the role of AhpC in providing resistance against RNS by using nitric oxide (NO) donor sodium nitroprusside (SNP) and Sin-1. Wild type *F*. *tularensis* LVS, the Δ*ahp*C mutant or the transcomplemented strains were exposed to varying concentrations of serially diluted SNP and Sin-1 for 1 and 3 hours and plated to determine the bacterial viability. The Δ*ahp*C mutant of *F*. *tularensis* LVS was found to be highly sensitive to both SNP (93.8 μg) and Sin-1 (0.1 and 0.05μg, respectively) as evidenced by marked reduction in viability after 1 and 3 hours of exposure to these compounds as compared to the wild type or the transcomplemented strain (Fig 4A and 4B). These results demonstrate that AhpC of *F*. *tularensis* LVS also plays an important role in providing resistance against RNS.

### The Δ*ahpC* mutant of *F*. *tularensis* LVS does not exhibit intramacrophage growth defect but is attenuated for virulence in mice

**Fig 5:**
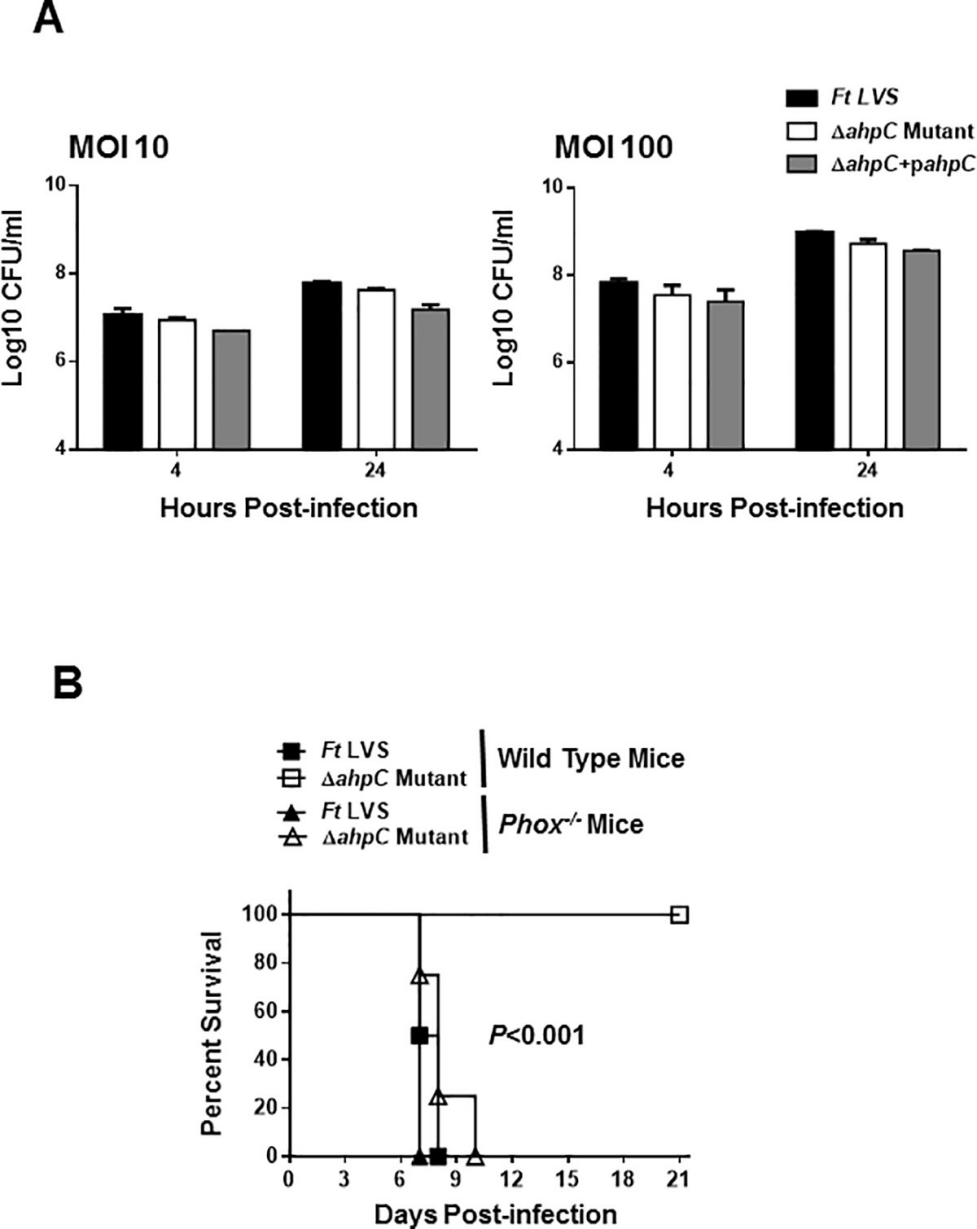
The Δ*ahpC* mutant of *F*. *tularensis* LVS does not exhibit intramacrophage growth defect, but is attenuated for virulence in mice. (A) Raw264.7 macrophages were infected with the *F*. *tularensis* (*Ft*) LVS, the Δ*ahpC* mutant or the transcomplemented strain (Δ*ahpC*+p*ahpC*) at 10 and 100 MOI (n = 3 biological replicates). The cells were lysed after 4 and 24 hrs of infection, serially diluted and plated on MH-chocolate agar plates for enumeration of bacterial CFU. The data are representative of three independent experiments conducted and are expressed as Log_10_ CFU/mL. (B) C57BL/6 and *phox*^*-/-*^ mice (*n* = 4 mice/group) were infected intranasally with 1x10^4^ CFUs of *F*. *tularensis* LVS or the Δ*ahpC* mutant and observed for mortality for a period of 21 days post-infection. The results are expressed as Kaplan-Meier survival curves, and the *p* values were determined using the Log-rank test. The comparison shown is between the wild type mice infected with *Ft* LVS and the Δ*ahpC* mutant.

The contribution of AhpC of *F*. *tularensis* LVS in intramacrophage survival was determined by macrophage gentamicin protection assay. Almost equal numbers of wild type *F*. *tularensis* LVS, the Δ*ahpC* mutant, and the transcomplemented bacteria invaded the cells at 4 hours post-infection. Nearly 2-fold fewer Δ*ahpC* mutant bacteria as compared to the wild type *F*. *tularensis* LVS were recovered from macrophages infected with 10 MOI. Similarly, after 24 hours of infection, 2-fold fewer Δ*ahpC* mutant bacteria (8.7±0.1 Log_10_ CFU/mL) as compared to the wild type *F*. *tularensis* LVS (8.9±0.0 Log_10_ CFU/mL) were recovered from the macrophages infected with 100 MOI. However, the fold-increase at 24 hours for both the wild type *F*. *tularensis LVS and the* Δ*ahpC* mutant remained similar. These results indicate that *ahpC* is not required for intramacrophage survival of *F*. *tularensis* LVS (Fig 5A).

We next examined the contribution of AhpC of *F*. *tularensis* LVS in virulence in mice. Since our preceding results indicated that the Δ*ahpC* mutant is highly sensitive to ROS, we also determined the contribution of NADPH oxidase-dependent ROS in clearance of Δ*ahpC* mutant of *F*. *tularensis* LVS by infecting *Phox*^*-/-*^ mice. These mice are defective in ROS generation. Wild-type C57BL/6 and *Phox*^*-/-*^ mice were infected intranasally with 1×10^4^ CFUs of either the wild-type *F*. *tularensis* LVS or the Δ*ahpC* mutant and observed for mortality for 21 days. 100% of wild type C57BL/6 mice infected with the Δ*ahpC* mutant survived the infection; while mice infected with similar doses of the wild type *F*. *tularensis* LVS succumbed to infection by day 8 post-infection, indicating that AhpC is required for virulence. On the other hand, 100% of *Phox*^*-/-*^ mice infected either with *F*. *tularensis* LVS, or the Δ*ahpC* mutant succumbed to infection indicating that NADPH-oxidase induced ROS is required for clearance of the Δ*ahpC* mutant (Fig 5B).

### AhpC of *F*. *tularensis* SchuS4 is a major antioxidant enzyme that protects against oxidative stress induced by superoxide-generating compounds

Previous studies conducted with mutants of *F*. *tularensis* LVS deficient in SodB, SodC, or KatG have reported that loss of only one antioxidant enzyme results in an enhanced sensitivity of *F*. *tularensis* LVS to oxidative stress, attenuated intramacrophage growth and virulence in mice [7,8,11]. The results obtained in this study with the Δ*ahpC* mutant of *F*. *tularensis* LVS also support this notion. On the contrary, the reported phenotype of the SchuS4 Δ*katG* mutant is quite different from that reported for the corresponding mutant of *F*. *tularensis* LVS [11]. Moreover, unlike *F*. *tularensis* LVS mutants, the Δ*katG*, Δ*sodC* and Δ*ahpC* mutants of *F*. *tularensis* SchuS4 retain their virulence in mice [10,11,16]. We next investigated to establish if AhpC is one of the major antioxidant enzymes of *F*. *tularensis* SchuS4 by determining the sensitivities of the Δ*ahpC*, Δ*sodC* and Δ*katG* mutants of *F*. *tularensis* SchuS4 to oxidants and RNS.

**Fig 6:**
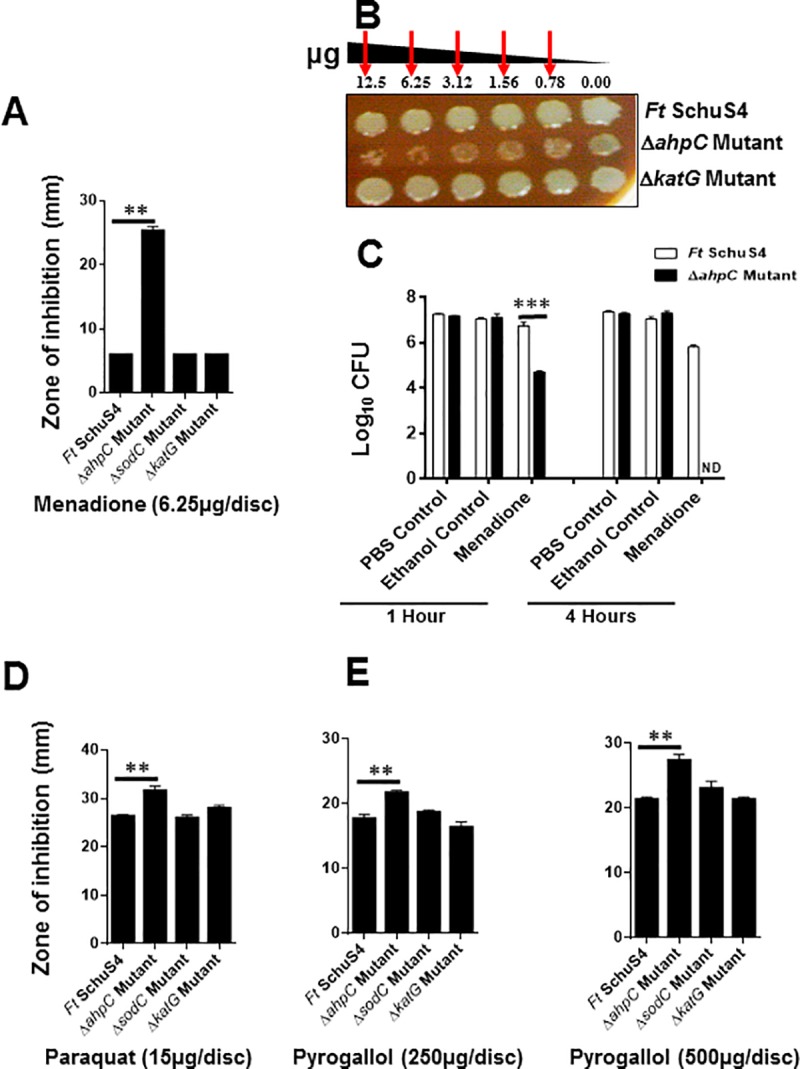
AhpC of *F*. *tularensis* SchuS4 is a major antioxidant enzyme that protects against oxidative stress induced by superoxide generating compounds. The sensitivities of the wild type *F*. *tularensis* (*Ft*) SchuS4, the Δ*ahpC*, Δ*sodC* and the Δ*katG* mutants of SchuS4 as determined by disc diffusion (A), spot assay (B) and bacterial killing assay (C) against superoxide-generating compound, menadione. The sensitivity of the indicated strains against paraquat (D) and pyrogallol (E) was determined using the indicated concentration of the compounds by disc diffusion assay. For the disc diffusion assays, the results are expressed as a zone of inhibition in millimeters and are expressed as Mean ± S.D. The red arrows in (B) indicate enhanced killing of the SchuS4 Δ*ahpC* mutant at the indicated concentrations of menadione. For bacterial killing assay (C) indicated bacterial strains were exposed to menadione (6.25μg/mL) and the bacterial numbers were enumerated after 1 and 4 hours of exposure. PBS, and ethanol required for suspension of menadione were used as controls. The data shown are representative of 2 independent experiments each conducted with 3 biological replicates and were analyzed by one-way ANOVA. **P<0.01; ***P<0.001.

Exposure of *F*. *tularensis* SchuS4, the Δ*ahpC*, Δ*sodC* and the Δ*katG* mutants to the superoxide-generating compound menadione revealed that the Δ*ahpC* mutant was extremely sensitive to menadione as evident by significantly enlarged zone of inhibition (25.3±1.1 mm) as compared to the wild type *F*. *tularensis* SchuS4, Δ*sodC* and Δ*katG* mutants (6.0±0.0 mm for all the three strains, respectively). No differences in sensitivity towards menadione were observed between the wild type *F*. *tularensis* SchuS4 or the Δ*sodC* and the Δ*katG* mutants (Fig 6A). We further confirmed these findings by performing spot- and bacterial killing assays. Results from the spot assays (Fig 6B) demonstrated that exposure to increasing concentrations of menadione resulted in reduced viability of the Δ*ahpC* mutant as compared to wild type *F*. *tularensis* SchuS4, or the Δ*katG* mutant. In another approach, equal numbers of wild type *F*. *tularensis* SchuS4 and the Δ*ahpC* mutant were exposed to menadione (6.25μg/mL) for 1 and 4 hours, diluted 10-fold, and the bacterial killing was determined. The results demonstrated that after 1-hour post-treatment with menadione, significantly lower numbers of the Δ*ahpC* mutant bacteria (4.7±0.1 Log_10_ CFU/mL) survived as compared to the wild type *F*. *tularensis* SchuS4 strain (6.7±0.3 Log_10_ CFU/mL). After 4 hours of treatment, no colonies of the Δ*ahpC* mutant were recovered, while the viability of the wild type *F*. *tularensis* SchuS4 was only reduced by 10-fold (5.8±0.1 Log_10_ CFU/mL). The viability of both *F*. *tularensis* SchuS4 and the Δ*ahpC* mutant were not affected in the PBS control or exposure to the volume of ethanol that was used to resuspend menadione (Fig 6C).

Exposure to paraquat resulted in a significantly larger zone of inhibition for the Δ*ahpC* mutant (31.67 ± 1.53 mm) as compared to the wild type *F*. *tularensis* SchuS4 (26.3±0.5 mm). However, treatment of Δ*sodC* (26.0±1.0 mm) and Δ*katG* (28.0±1.0 mm) mutant strains with paraquat did not show any enhanced sensitivity as compared with the wild type *F*. *tularensis* SchuS4 (Fig 6D). Disc diffusion assays using pyrogallol (250 and 500μg/disc) displayed similar results, with Δ*ahpC* mutant strain showing a significantly enlarged zone of inhibition (21.6±1.5 and 27.3±1.5 mm, respectively) as compared to the wild type *F*. *tularensis* SchuS4 strain (17.6±1.1 and 21.3±0.5 mm, respectively). Further, similar to paraquat, the Δ*sodC* (18.6±0.5 and 23.0±2.0 mm, respectively) and Δ*katG* (16.3±1.5 and 21.3±0.5 mm, respectively) mutant strains did not show any increased sensitivity to pyrogallol when compared with the wild type *F*. *tularensis* SchuS4 (Fig 6E). Collectively, these results indicate that AhpC of *F*. *tularensis* SchuS4 is primarily responsible for providing resistance against oxidative stress induced by superoxide radicals. These results also demonstrate that both the SodC and KatG are dispensable, as the loss of these antioxidant enzymes do not alter the sensitivities of the Δ*sodC* and Δ*katG* mutants to superoxide-generating compounds and remain similar to the wild type *F*. *tularensis* SchuS4 strain.

### AhpC of *F*. *tularensis* SchuS4 protects against oxidative stress induced by peroxides

**Fig 7:**
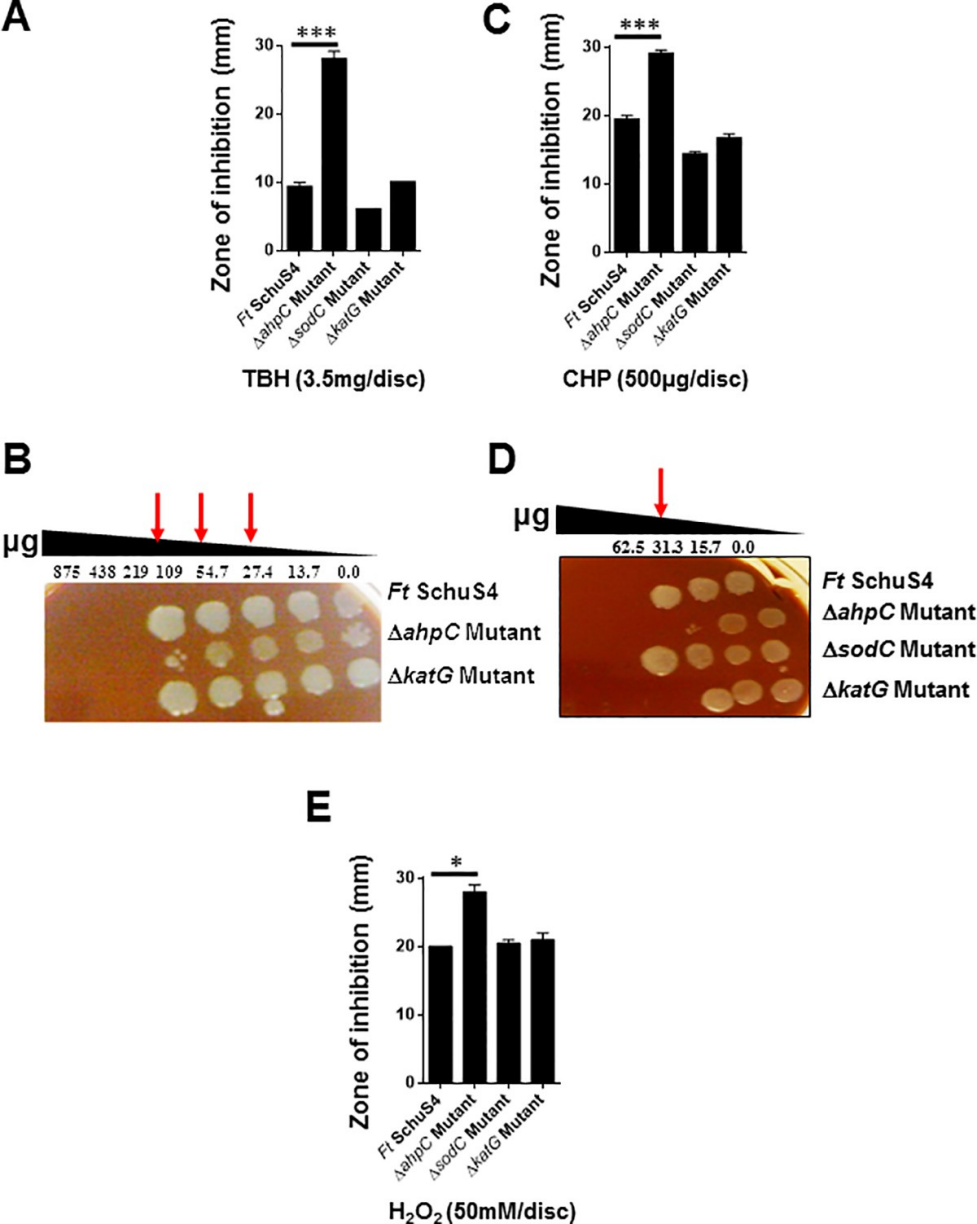
AhpC of *F*. *tularensis* SchuS4 protects against oxidative stress induced by peroxides. The sensitivities of the wild type *F*. *tularensis* (*Ft*) SchuS4, the Δ*ahpC*, Δ*sodC* and the Δ*katG* mutants of SchuS4 as determined by disc diffusion assays against tert-butyl hydroperoxide (TBH) (A), cumene hydroperoxide (CHP) (C) and H_2_O_2_ (E), and by spot assays against TBH (B) and CHP (D). For the disc diffusion assays, the results are expressed as zone of inhibition in millimeters and are expressed as Mean ± S.D. The red arrows in (B and D) indicate enhanced killing of the Δ*ahpC* mutant at the indicated concentrations of the compounds. The data shown are representative of two independent experiments each conducted with 3 biological replicates and were analyzed by one-way ANOVA, and *p* values were recorded. *P<0.05; ***P<0.001.

Disc diffusion assays using peroxides TBH, CHP and H_2_O_2_ exhibited results similar to those observed following treatment with superoxide-generating compounds. Exposure of Δ*ahpC* mutant to 3.5mg/disc of TBH demonstrated a significantly larger zone of inhibition (28.00 ± 2.0 mm) as compared to the wild type *F*. *tularensis* SchuS4 strain (9.3±1.1mm) (Fig 7A). However, the Δ*sodC* mutant strain (6.0±0.0mm) was observed to be more resistant to TBH than the wild type SchuS4 strain. The susceptibility of the Δ*katG* mutant (10.0±0.0 mm) to TBH treatment remained similar to that observed for the wild type *F*. *tularensis* SchuS4. The spot assay demonstrated similar results as observed for the disc diffusion assays; the Δ*ahpC* mutant was more sensitive to increasing concentrations of TBH than the wild type *F*. *tularensis* SchuS4 strain. However, the sensitivity of the Δ*katG* mutant remained similar to that observed for the wild type *F*. *tularensis* SchuS4 strain (Fig 7B).

Exposure of wild type *F*. *tularensis* SchuS4 and the Δ*ahpC*, Δ*sodC* and the Δ*katG* mutants to 500μg/disc of CHP demonstrated that the Δ*ahpC* mutant was significantly more sensitive to the compound (29.0 ± 1.0 mm) as compared to the wild type *F*. *tularensis* SchuS4 (19.3 ± 1.1 mm). The sensitivity of the Δ*katG* mutant to CHP (16.6±1.5 mm) remained similar to that observed for the wild type *F*. *tularensis* SchuS4. On the other hand, similar to that observed for TBH, the Δ*sodC* mutant was also more resistant to CHP (14.3±0.6mm) as compared to the wild type *F*. *tularensis* SchuS4 strain (Fig 7C) as determined by the disc diffusion assays as well as by spot assay (Fig 7D). The Δ*ahpC* mutant demonstrated higher sensitivity to 50mM/disc of H_2_O_2_ as indicated by a greater zone of inhibition (28.00±1.4 mm) compared to the wild type *F*. *tularensis* SchuS4 (20.0±0.0 mm) (Fig 7E), whereas, the sensitivities of the Δ*sodC* and Δ*katG* mutants remained similar to those observed for the wild type SchuS4 strain (20.5±0.7 and 21.0±1.1 mm, respectively). Collectively, these results indicate that the requirement of AhpC for resistance against oxidative stress induced by superoxide radicals and peroxides.

### Exposure to NO-generating compounds results in the enhanced killing of Δ*ahpC* mutant of *F*. *tularensis* SchuS4

**Fig 8:**
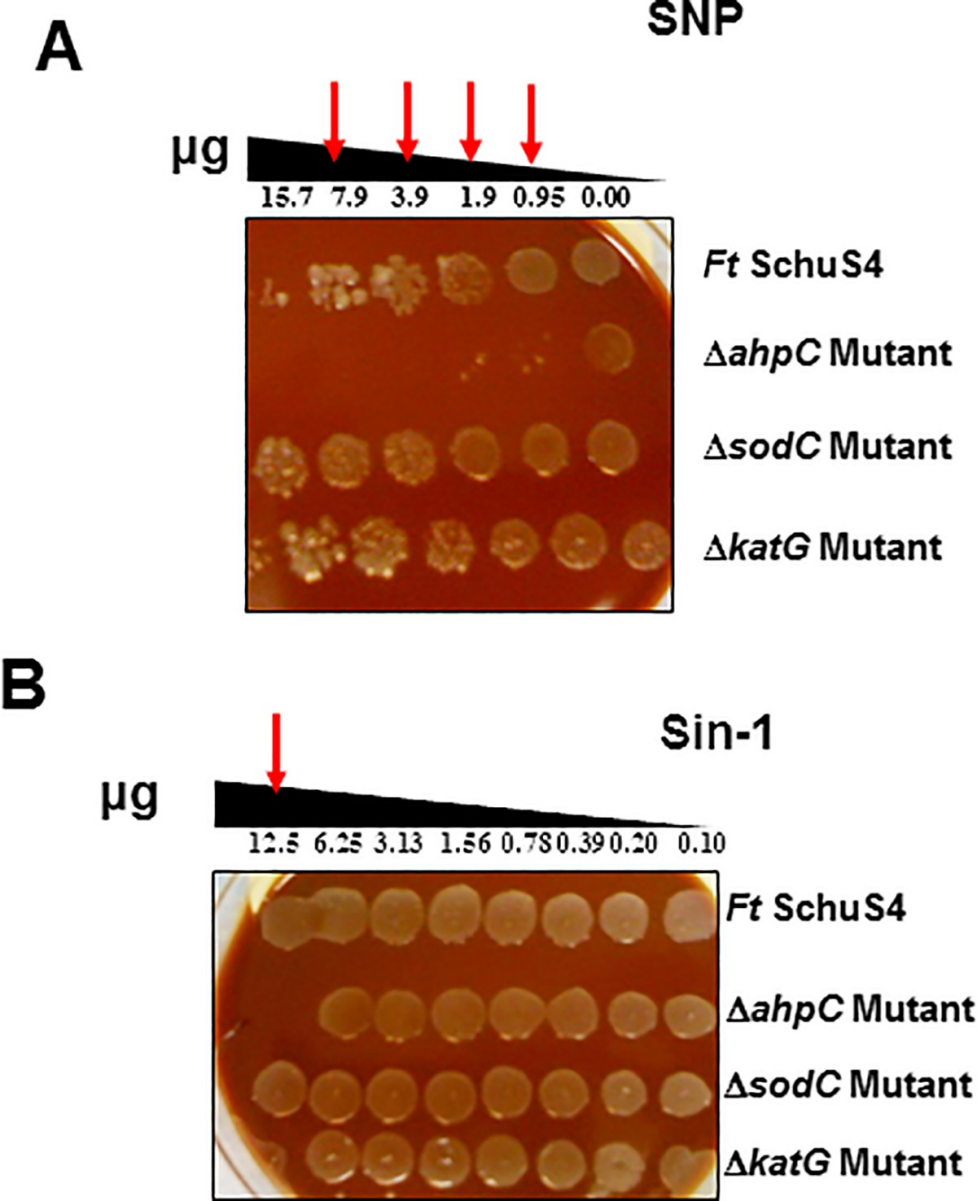
Exposure to nitric oxide generating compounds results in enhanced killing of the Δ*ahpC* mutant of *F*. *tularensis* SchuS4. Spot assays were performed using (A) sodium nitroprusside (SNP) and (B) Sin-1. Wild type *F*. *tularensis* (*Ft*) SchuS4, the Δ*ahpC*, Δ*sodC* and the Δ*katG* mutants of SchuS4 were exposed to serially diluted compounds for 1 hour and spotted on MH-chocolate agar plates. The red arrows indicate enhanced killing of the Δ*ahpC* mutant at the indicated concentrations of the compounds. The results shown are representative of two independent experiments conducted.

We next investigated the role of *F*. *tularensis* SchuS4 antioxidants in providing resistance to RNS. Results of this assay demonstrated that the Δ*ahpC* mutant was highly sensitive to increasing concentrations of SNP and SIN-1 (Fig 8A and 8B) as compared to the wild type *F*. *tularensis* SchuS4 or the Δ*katG* mutant. However, the Δ*sodC* mutant showed enhanced resistance to SNP as compared to the wild type *F*. *tularensis* SchuS4 strain (Fig 8A). These results demonstrate that AhpC in addition to ROS also protects *F*. *tularensis* SchuS4 against RNS.

### The Δ*ahpC* mutant of *F*. *tularensis* SchuS4 is attenuated for intramacrophage growth

**Fig 9:**
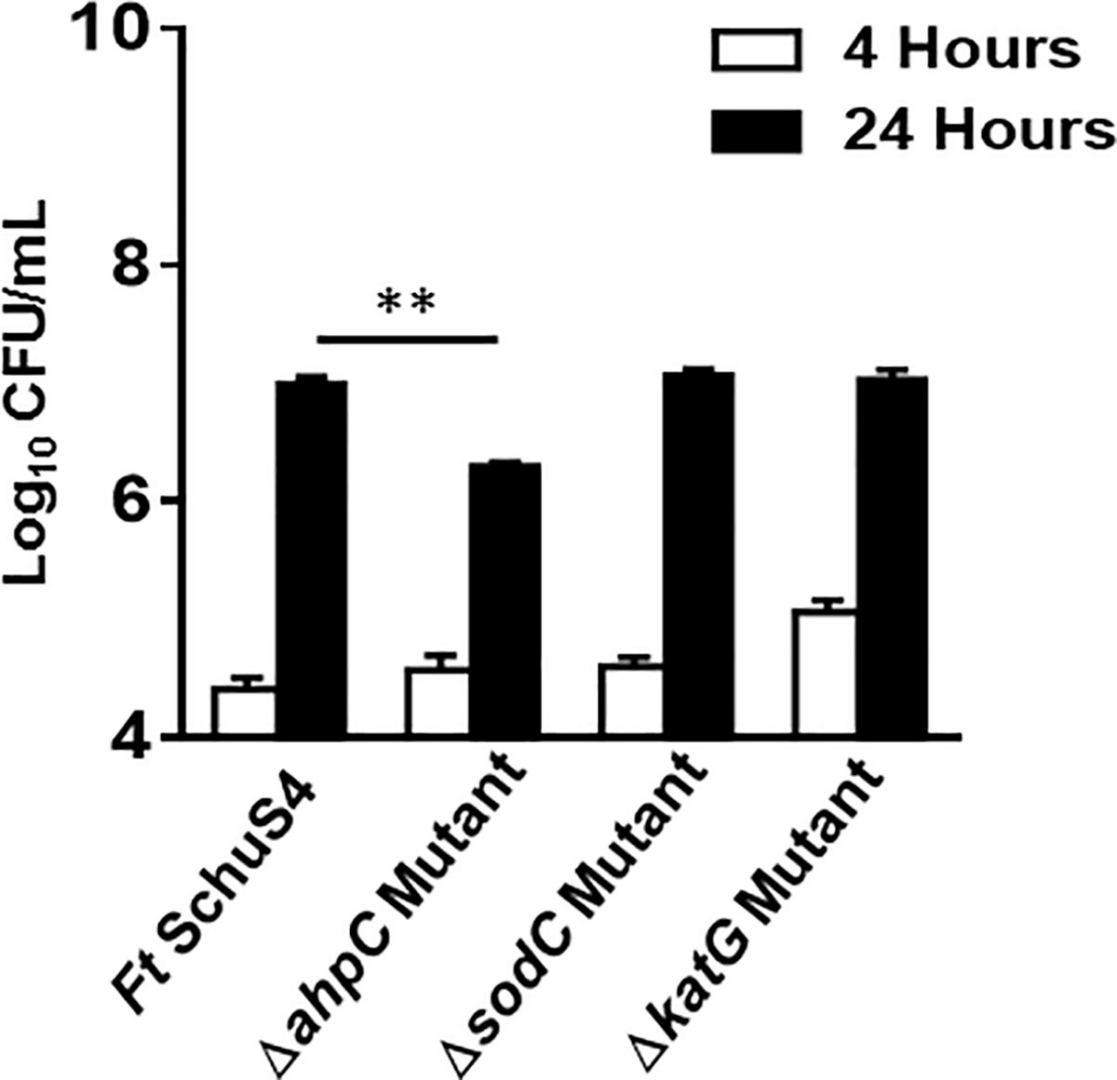
The Δ*ahpC* mutant of *F*. *tularensis* SchuS4 is attenuated for intramacrophage growth. Raw264.7 macrophages were infected with the wild type *F*. *tularensis* (*Ft*) SchuS4, the Δ*ahpC*, Δ*sodC* and the Δ*katG* mutants of SchuS4 at 100 MOI (n = 3 biological replicates). The cells were lysed after 4 and 24 hrs of infection, serially diluted and plated on MH-chocolate agar plates for enumeration of bacterial CFU. The data are expressed as Log_10_ CFU/mL. The data shown are representative of two independent experiments each conducted with 3 biological replicates and were analyzed by one-way ANOVA, and *p* values were recorded. **P<0.01.

To determine the role of *F*. tularensis SchuS4 antioxidants in intramacrophage survival, we infected Raw264.7 macrophages with the wild type *F*. *tularensis* SchuS4 and the Δ*ahpC*, Δ*sodC* and the Δ*katG* mutants at an MOI of 100 and lysed the cells 4 and 24 hours post-infection. It was observed that significantly lower numbers of the Δ*ahpC* mutant bacteria (6.2±0.1 Log_10_ CFU/mL) were recovered from Raw264.7 cells at 24 hours post-infection as compared to the wild type *F*. *tularensis* SchuS4 strain (6.9±0.1 Log_10_ CFU/mL). Higher numbers of Δ*katG* mutant bacteria were taken up by the macrophages as compared to the wild type *F*. *tularensis* SchuS4, the Δ*ahpC*, and the Δ*sodC* mutants at 4 hours post-infection. However, both Δ*katG* and Δ*sodC* mutants survived and replicated similarly to the wild type *F*. *tularensis* SchuS4 strain and equal numbers of bacteria (7.0±0.1 and 7.0±0.2 Log_10_ CFU/mL, respectively) were recovered at 24 hours post-infection. The wild type *F*. *tularensis* SchuS4 and the Δ*sodC* bacteria showed a 25-fold increase at 24 hours post-infection than those recovered from macrophages after 4 hours of infection. The Δ*katG* mutants exhibited 20-fold increase; while the Δ*ahpC* mutants increased by 17-fold at 24 hours post-infection. These results demonstrate that AhpC contributes to intramacrophage growth of *F*. *tularensis* SchuS4 (Fig 9).

## Discussion

*Francisella tularensis* during its intracellular residence encounters a number of oxidative and nitrosative stresses. To overcome these, *F*. *tularensis* has evolved a multitude of mechanisms. *Francisella* counters the phagocyte induced oxidative stress by relying on two divergent approaches; neutralize the ROS/RNS produced by the phagocytic cells and inhibit the assembly of NADPH oxidase [21]. The roles of the primary antioxidant enzymes SodB, SodC and KatG of *F*. *tularensis* LVS have been characterized in previous studies [7,11,22]. It has been reported that these antioxidant enzymes are required for resistance of *F*. *tularensis* LVS against oxidative stress, survival in IFN-γ stimulated macrophages and virulence in mice [7,8,11]. On the contrary, it has been reported that KatG of *F*. *tularensis* SchuS4 although provides some degree of resistance against H_2_O_2_, is neither required for intramacrophage survival nor virulence in mice [11]. Similarly, both SodC and AhpC of *F*. *tularensis* SchuS4 are not required for virulence in mice [16]. Very fragmentary information is available regarding the role of both SodC and AhpC [16] of *F*. *tularensis* SchuS4, and none related to AhpC of *F*. *tularensis* LVS. This study investigated the role of AhpC in oxidative and nitrosative stress resistance of *F*. *tularensis* LVS and SchuS4.

Results from this study demonstrate that AhpC plays a role in the protection of *Francisella* against the oxidative and nitrosative stresses. Furthermore, it was observed that loss of *ahpC* in *F*. *tularensis* LVS is not associated with any intramacrophage growth defect in unstimulated naïve macrophages, but the Δ*ahpC* mutant is attenuated for virulence in mice; a phenotype consistent with the Δ*sodC* and Δ*katG* mutants of *F*. *tularensis* LVS (8, 11). These findings indicate that antioxidant enzymes of *F*. *tularensis* LVS act independently and that loss of one enzyme is not compensated by other antioxidant enzymes in response to oxidative or nitrosative stresses. The SchuS4 Δ*ahpC* mutant showed higher sensitivities towards superoxide-generating compounds and peroxides. However, unlike *F*. *tularensis* LVS mutants, the sensitivities of both the SchuS4 Δ*sodC* and Δ*katG* mutants towards superoxide-generating compounds as well as peroxides remained similar to the wild type *F*. *tularensis* SchuS4. These observations indicate that *F*. *tularensis* SchuS4 AhpC serves as a major antioxidant enzyme in providing resistance against oxidative stresses. Furthermore, the SchuS4 Δ*ahpC* mutant was found to be attenuated for intramacrophage growth indicating that AhpC in *F*. *tularensis* SchuS4 unlike LVS, play a role in overcoming the oxidative stress intracellularly.

The Δ*ahpC* mutants of both *F*. *tularensis* LVS and SchuS4 exhibited an unusually high sensitivity towards RNS generating compounds SNP and Sin-1. SNP exerts its bactericidal effect by releasing NO which can either be oxidized or reduced to generate highly reactive and microbicidal RNS [23,24]. RNS reacts with cellular thiols, lipids and metals to inhibit metabolism, damage cell membranes and DNA [25]. NO also reacts with superoxide anion to produce highly reactive peroxynitrite anion (ONOO^-^) [26] which is subsequently decomposed into potent microbicidal reactive nitrogen intermediates [26,27]. Sin-1 generates ONOO^-^ by producing both NO and superoxide anions. ONOO^-^ is required for macrophage-dependent killing of *F*. *tularensis* [28]. However, our previous studies have shown that neither the Δ*sodC* nor the *sodB*Δ*sodC* mutants of *F*. *tularensis* LVS exhibit enhanced sensitivities towards NO or preformed ONOO^-^ under cell-free growth conditions [22]. Similarly, the viability of the *F*. *tularensis* LVS Δ*katG* mutant is only partially affected; while the viability of the SchuS4 Δ*katG* mutant remains similar to its parental wild type strain upon exposure to Sin-1 [11]. These observations indicate that superoxide dismutases and catalase of *F*. *tularensis* LVS and SchuS4 are primarily involved in scavenging ROS, but do not effect RNS. On the other hand, enhanced sensitivities of Δ*ahpC* mutants of both *F*. *tularensis* LVS and SchuS4 towards SNP and Sin-1 observed in this study demonstrate that AhpC contributes to resistance against nitrosative stresses.

Majority of Gram-negative bacteria encode AhpC belonging to 2-Cys peroxiredoxins to protect bacteria from ROS and RNS induced cell damage [29]. A conserved peroxidatic cysteine in AhpC reacts with H_2_O_2_ or organic peroxides to form sulfenic acid and then subsequently release water or the corresponding alcohols. The oxidized AhpC is reduced and regenerated by an NADH-dependent oxidoreductase AhpF [29]. The AhpC of *F*. *tularensis* differs from other members of the peroxiredoxin family of proteins. *F*. *tularensis* AhpC is a 1-Cys peroxiredoxin containing a conserved peroxidatic cysteine; however, it lacks the resolving cysteine as well as the reducing partner AhpF. Similar to *F*. *tularensis*, AhpC in mycobacteria protects against RNS and hydroperoxides [30]. Mycobacterial AhpC catalyzes the conversion of ONOO^-^ to nitrite very rapidly and prevents its spontaneous decomposition into highly microbicidal nitrogen dioxide and hydroxyl radicals [31]. However, unlike *F*. *tularensis*, the *M*. *tuberculosis* AhpC is a 3-Cys peroxiredoxin containing the peroxidatic cysteine, the putative resolving cysteine and the third cysteine with unknown catalytic role [32]. The peroxidatic cysteine of the mycobacterial AhpC attacks ONOO^-^ and gets oxidized to cysteine sulfenic acid residues; while the resolving cysteine completes the catalytic cycle. A thioredoxin-like protein known as AhpD reduces the oxidized AhpC in mycobacteria [31]. The mechanisms through which the AhpC of *F*. *tularensis* neutralizes ONOO^-^ in the absence of a resolving cysteine and how AhpC is regenerated in *F*. *tularensis* in the absence of AhpD/AhpF homologs is yet to be elucidated.

Collectively, this study highlights differences in antioxidant defense mechanisms of *F*. *tularensis* LVS and SchuS4 and their abilities to counter oxidative and nitrosative stresses. Nearly 4–5 times the concentration of oxidants and RNS generating compounds used for *F*. *tularensis* LVS were required to get tangible results with *F*. *tularensis* SchuS4 mutants. One hundred percent of the wild type *F*. *tularensis* LVS bacteria were killed when the concentrations of the compounds used in assays with *F*. *tularensis* SchuS4 were applied. However, these concentrations either did not affect or only moderately affected the viability of *F*. *tularensis* SchuS4. To conclude, our results demonstrate that AhpC of *F*. *tularensis* LVS confers resistance against a wide range of ROS and RNS, and serves as a virulence factor. In highly virulent *F*. *tularensis* SchuS4 strain, AhpC serves as a key antioxidant enzyme and contributes to its robust oxidative and nitrosative stress resistance, and intramacrophage survival. It also becomes evident from these results that *F*. *tularensis* SchuS4 can compensate for the loss of KatG and SodC with other antioxidant enzymes, but may not do so when AhpC is absent. The results from this study further indicate that differences in virulence attributes of *F*. *tularensis* LVS and SchuS4 may be due to the inherent differences in their antioxidant defense mechanisms.

## Supporting information

S1 Fig The sensitivities of the wild type F. tularensis (Ft) LVS, the ΔsodC mutant, and the ΔkatG mutants. The sensitivities of *Ft* LVS, Δ*sodC* mutant, and the Δ*katG* mutants were determined by disc diffusion and spot assays against superoxide-generating compounds menadione (A and B), TBH (C and D), and CHP (E and F). For disc diffusion assays, the results are expressed as a zone of inhibition in millimeters obtained using the indicated concentrations of the compounds and are expressed as Mean ± S.D. of triplicate samples. All the results shown are representative of 3 independent experiments conducted. The *p* values were determined by one-way ANOVA and a *p*-value of <0.05 is considered statistically significant. *p<0.05; **p<0.01, ***p<0.001.
(TIF)
