# Supplementary figures and images for: Role of peroxiredoxin of the AhpC/TSA family in antioxidant defense mechanisms of Francisella tularensis
Source: PLoS One. 2019 Mar 14;14(3):e0213699. doi: 10.1371/journal.pone.0213699 (PMC6417708; doi:10.1371/journal.pone.0213699)

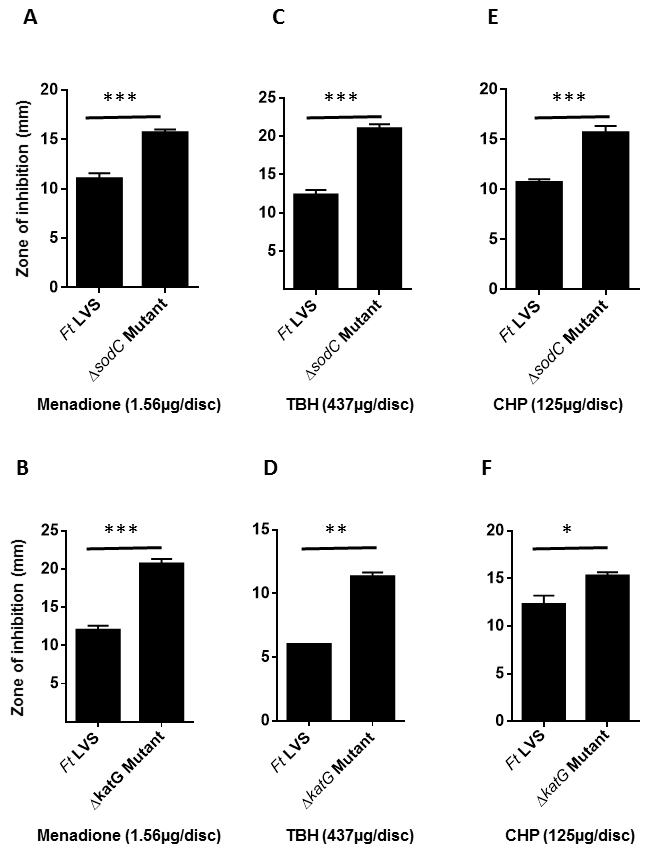

Supplement: S1 Fig — The sensitivities of Ft LVS, ΔsodC mutant, and the ΔkatG mutants were determined by disc diffusion and spot assays against superoxide-generating compounds menadione (A and B), TBH (C and D), and CHP (E and F). For disc diffusion assays, the results are expressed as a zone of inhibition in millimeters obtained using the indicated concentrations of the compounds and are expressed as Mean ± S.D. of triplicate samples. All the results shown are representative of 3 independent experiments conducted. The p values were determined by one-way ANOVA and a p-value of <0.05 is considered statistically significant. *p<0.05; **p<0.01, ***p<0.001. (TIF) [file pone.0213699.s001.tif]
